# The Current Role of Antiangiogenics in Colorectal Cancer

**DOI:** 10.3390/ijms262311605

**Published:** 2025-11-29

**Authors:** Debora Basile, Paola Di Nardo, Maria Grazia Daffinà, Carla Cortese, Jacopo Giuliani, Giuseppe Aprile

**Affiliations:** 1 Unit of Medical Oncology, San Giovanni Di Dio Hospital-Crotone, 88900 Crotone, Italy; deborabasile1090@gmail.com (D.B.); oncologacarlacortese@alice.it (C.C.); 2 Department of Medical Oncology, Centro di Riferimento Oncologico (CRO), IRCCS, 33081 Aviano, Italy; pidinardo@gmail.com; 3 Unit of Medical Oncology, Giovanni Paolo II Hospital–Lamezia, ASP Catanzaro, 88046 Lamezia Terme, Italy; mariagraziadaffy@libero.it; 4 Department of Oncology, Mater Salutis Hospital, Az. ULSS 9 Scaligera, 37045 Legnago, Italy; jacopo.giuliani@aulss9.veneto.it; 5 Department of Oncology, Azienda Sanitaria Universitaria Friuli Centrale, 33100 Udine, Italy

**Keywords:** antiangiogenics, colorectal cancer, anti-VEGF, CRC

## Abstract

Colorectal carcinoma (CRC) represents the third most common cancer worldwide. Approximately 20% of patients present with metastatic disease at diagnosis, and 30–50% experience disease recurrence over time. For metastatic CRC (mCRC), the standard treatment consists of chemotherapy combined with a targeted agent based on molecular profile, such as RAS, BRAF, and MSI status. Anti-angiogenic drugs, which inhibit the formation of new blood vessels, have an established role in the management of mCRC. Mounting evidence highlights the critical interplay among angiogenesis, hypoxia, and the immune response in tumor progression. These insights have paved the way for testing novel combinations and molecules to control cancer progression. In particular, combining anti-angiogenic agents with immune checkpoint inhibitors has shown promise in improving outcomes for mCRC patients. Among emerging therapies, the novel anti-angiogenic agent fruquintinib has recently demonstrated clinical efficacy in the treatment of mCRC. Based on the data discussed in the present narrative review, the therapeutic landscape of mCRC appears poised for significant evolution in the near future. While numerous challenges and unanswered questions remain, the emergence of innovative therapeutic combinations and agents provides a promising opportunity for improving patient outcomes in mCRC.

## 1. Introduction

Colorectal cancer (CRC) is the third most common cancer and the second leading cause of death by cancer worldwide [1]. For unresectable, advanced, or metastatic CRC (mCRC), the backbone of any therapeutic strategy is often represented by systemic treatment—chemotherapy associated with a targeted agent. There are multiple options for first-line therapy, depending on the biological molecular profile of the cancer (such as RAS, BRAF, and MSI status) and the patient’s individual characteristics.

Due to their increased metabolism, cancer cells require an increased availability of nutrients and oxygen, which is dependent on the generation of new blood vessels (angiogenesis); solid tumors have, therefore, acquired the ability to induce angiogenesis (angiogenic switch); among other mechanisms key to this process is the increased secretion of pro-angiogenic factors, such as VEGFs [2,3]. Hence, the development of angiogenic-targeting drugs has been increasingly relevant in the past few decades.

The first available anti-angiogenetic drug was bevacizumab, which inhibits the activation of VEGF signaling pathways through binding VEGF-A. In 2004, Hurwitz et al. demonstrated survival improvement by adding bevacizumab to FOLFIRI in the treatment of mCRC; hence, bevacizumab was approved for the treatment of mCRC in 2004 and 2005 in the United States (US) and the European Union (EU), respectively [4]. In August 2012, following the results from the VELOUR trial, the FDA approved ziv-aflibercept; this is a humanized recombinant fusion protein that functions as a VEGF inhibitor [5]. In September of the same year, regorafenib, a multi-kinase inhibitor that also has antiangiogenetic activity, was approved for advanced mCRC [6]. In 2013, the EMA also granted approval first for ziv-aflibercept and then for regorafenib. Lastly, in April 2014, ramucirumab, a recombinant human monoclonal IgG1 antibody that binds to the VEGF-R2, was also approved by the FDA (and by the EMA in December of the same year), based on the results of the RAISE trial [7].

Since then, no new antiangiogenetic drug has been approved, possibly owing to immunotherapy’s rise in prominence over the last few years. Still, the relevance of antiangiogenetic drugs in current clinical practice for the treatment of mCRC is undeniable; in this review, we will try to summarize the role of angiogenesis in pathophysiology, the clinical role of antiangiogenetic drugs, and potential developments for the future in mCRC.

## 2. From Benchside to Bedside

### 2.1. Role of Angiogenesis in Pathophysiology of CRC

The term “angiogenesis” was first proposed by Folkman over 50 years ago [8]. Angiogenesis is the formation of new capillaries out of pre-existing blood vessels under the regulation of growth and inhibitor factors [9]. This process is a multistep mechanism involving the interplay between many biological components, such as several cell types such as endothelial cells, tumor cells, stromal cells, immune-infiltrating cells (e.g., neutrophils and tumor-associated macrophages, “TAMs”), soluble angiogenic factors, and extracellular matrix (ECM) components [10,11,12]. Angiogenesis is intricate and primarily consists of four distinct consequential steps: (I) degradation of basement membrane glycoproteins and other components of the ECM surrounding the blood vessels by proteolytic enzymes; (II) endothelial cell (EC) activation and migration; (III) EC proliferation; and (IV) ECs transforming into tube-like structures, forming capillary tubes, and developing into novel basement membranes [12]. This process occurs in many physiological conditions through a balance between pro- and anti-angiogenic factors, as well as between multiple signal transduction pathways [9]; however, aberrant angiogenesis is a key process in cancer growth, invasion, and metastasis [12].

Neo-angiogenesis is primarily involved in CRC tumorogenesis, with two main regulators, hypoxia factor-1α (HIF-1α) and vascular endothelial growth factor (VEGF). HIF-1α is secreted by the cancer cell under hypoxic conditions and synergically acts with other pro-angiogenic molecules, such as VEGF, placental growth factor (PIGF), or angiopoietins. This factor affects a great variety of signal transduction pathways, including the up-regulation of the VEGF signaling cascade [11,13]. VEGF family includes a group of glycoproteins (VEGF-A, VEGF-B, VEGF-C, and VEGF-D) that, together with PlGF, interact with three VEGF receptors (VEGFR-1, -2, and -3) and two neuropilin co-receptors (NRP1, NRP2). VEGFA is produced by most cells in the body, but is up-regulated in hypoxic conditions [14]. In tumors, VEGF is produced by hypoxic tumor cells, ECs, and infiltrating myeloid cells, also known as the aforementioned TAMs [14,15].

The VEGF-A gene consists of eight exons on chromosome 6, with splice variants forming different isoforms, among which VEGFA165 is the most biologically active [16]. Most targeted cancer therapies act by inhibiting VEGF-A splice isoforms that promote microvessel growth, which is responsible for the most advanced and aggressive forms of the disease. Notably, the VEGF-A isoform balance, which is controlled by messenger ribonucleic acid (mRNA) splicing, coordinates angiogenesis [16].

VEGF-B and VEGF-A share the same architecture and activity, with a key role in carcinogenesis and blood vessels persistence, rather than an “angiogenic” factor, under stress conditions [17].

The VEGF pathway is up-regulated by several growth factors, including epidermal growth factor (EGF), platelet-derived growth factors (PDGFs), hepatocyte growth factor (HGF), and other cytokines [18].

VEGFRs are tyrosine kinase receptors (RTKs) found primarily in vascular endothelial cells [19]. The binding of the glycoprotein to its receptor results in the initiation of a sequence of events that ultimately leads to the formation of new vessels. The ligation of VEGF-A with VEGFR-2 is the most important step in the activation of angiogenesis in CRC [11,20]. This binding triggers multiple signaling networks that result in endothelial cell survival, migration, mitogenesis, differentiation, vascular permeability and vascular inflammation, vasodilatation, alteration of gene expression, and activation of the Ras pathway [9,14,18,20].

The role of VEGFR-1 is more complex and not fully understood. A soluble form of VEGFR-1 can prevent the binding between VEGF-A and VEGFR-2, which, in turn, inhibits the activation of the downstream pathway. However, VEGFR-1 is also involved in tumor-associated angiogenesis [21]. The third receptor, VEGFR-3, is involved in lymphangiogenesis, binding VEGF-C and VEGF-D [20]. Regarding VEGF-D expression, it has been associated with regional lymph node metastasis [9].

Likewise, PlGF acts by regulating endothelial and mural cell proliferation, as well as by engaging other pro-angiogenic cells and molecules. Moreover, it has a structural homology with VEGF-A, and thus, it interacts with VEGFR-1 downstream signaling [9]. This binding unleashes TAMs into the tumor bed, where they exert their immune-suppressive and pro-angiogenic function [22].

Microvascular density (MVD) is another important indicator used as a surrogate marker of tumoral angiogenesis and has been proposed to identify patients at a high risk of recurrence. MVD assessment is the most used technique to quantify the degree of neovascularization of the tumor [11,23]. MVD appears to increase during the evolutionary events in the sequence from normal mucosa to adenoma and from adenoma to cancer in CRC patients [24]. Since MVD is a biomarker for the quantification of angiogenesis, the question arises whether it can be used as a predictor biomarker for treatment with the antiangiogenic agents [25,26].

### 2.2. The Crosstalk Between Angiogenesis and Immune System

Tumor-associated blood vessels show an abnormal structure and a dysfunctional vascular network that modulate the expression of pro-inflammatory and co-stimulatory molecules; thus, they contribute to the permeability of immune-suppressing cells. Hence, cancer cells, through the overexpression of VEGFA, acquire the ability to affect, in turn, the immune and blood texture, shifting the tumor microenvironment (TME) towards an immune-suppressed microenvironment [11,27]. Within TME, immune and endothelial cells interact continuously with each other in a tight and mutual crosstalk, developing a dynamic context. Immune cells directly affect the phenotypes and functions of cancer vessels through various cytokines [28].

Innate immune cells, such as mature dendritic cells and M1-tumor-associated macrophages (TAM), produce cytokines (IFN-α, IL-12, IL-18, or TNF-α) and chemokines (CXCL9, CXCL10, or CCL21) that suppress tumor angiogenesis. Meanwhile, adaptive immune cells, such as CD8+ T cells and T helper 1 cells (TH1), secrete IFN-γ, a potent cytokine that inhibits angiogenesis and induces vascular normalization in TME [28].

The increased production of VEGF inhibits differentiation and maturation of monocytes into dendritic cells (DCs), impacting the antigen presentation, inhibiting nuclear factor (NF-kB), up-regulating programmed death-ligand 1 (PD-L1) on DCs, and, finally, inducing T cell suppression. Namely, VEGF inhibits progenitor cell differentiation into CD4+ and CD8+ lymphocytes, T cells’ proliferation, and increases T cells’ exhaustion by up-regulating PD-L1, cytotoxic T-lymphocytes associated protein 4 (CTLA4), TIM3, and LAG3 in T lymphocytes [29,30,31].

Neo-angiogenesis enhances the intra-tumoral pressure and decreases the endothelial intracellular adhesion molecule-1, such as the vascular cell adhesion molecule (VCAM-1) [32,33,34]. Likewise, hypoxia and acidosis induce the up-regulation of immune-suppressive chemokines that attract regulatory T cells (Treg), myeloid-derived suppressor cells (MDSCs) at the tumor site, polarize macrophages towards the M2-like phenotype, and reduce the extravasation of tumor-infiltrating cells (TILs). Moreover, pro-tumorigenic triggers are enhanced by the expression of FasL on tumor endothelial cells, which in turn, inhibits the proliferation of TCD8+ effector cells’ bed, allowing tumor spreading and resistance to anti-cancer treatment [35,36,37].

**Figure 1:**
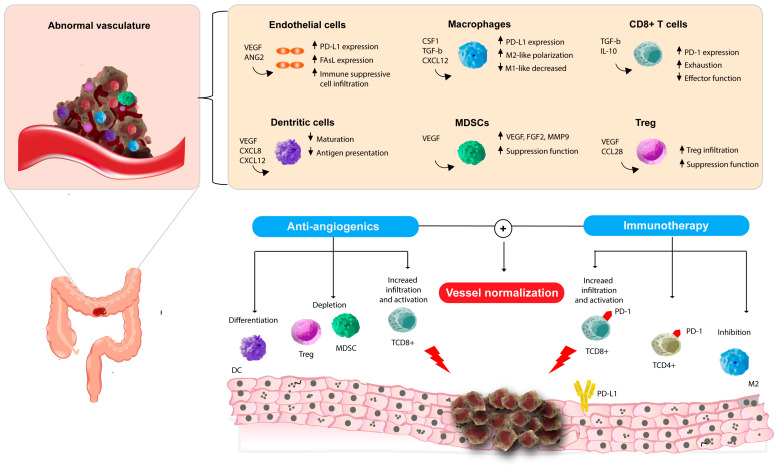
Interplay between angiogenesis, the immune system, and vessel normalization in colorectal cancer (the Figure was originally created by the authors with Adobe Illustrator CC 2015-version 19.0.0. Abnormal tumor vasculature recruits immune-suppressive cells by altering the function of endothelial cells (ECs), macrophages, dendritic cells, myeloid-derived suppressor cells (MDSCs), CD8^+^ T cells, and regulatory T cells (Tregs). Anti-angiogenic agents can normalize tumor vasculature, reduce hypoxia, and reestablish the immune microenvironment, particularly of CD8 T cells, while decreasing immune-suppressive cells. When combined with immunotherapy, this synergy enhances vessel normalization, T cell activation, and antitumor immunity. Abbreviations: ANG2 = Angiopoietin-2; CCL28 = C-C motif chemokine ligand 28; CSF1 = colony-stimulating factor 1; CXCL8/12 = C-X-C motif chemokine ligand 8/12; DC = dendritic cell; FGF2 = fibroblast growth factor 2; FAsL = Fas Ligand; IL-10 = Interleukin 10; MDSC = myeloid-derived suppressor cell; MMP9 = matrix metalloproteinase 9; M2 = M2-like macrophage; PD-1 = programmed cell death protein 1; PD-L1 = programmed death-ligand 1; TCD4^+^ = CD4^+^ T cell; TCD8^+^ = CD8^+^ T cell; TGF-b = transforming growth factor beta; Treg = Regulatory T cell; and VEGF = vascular endothelial growth factor.

The use of anti-angiogenetic treatments reverses the immunosuppressive effect created by VEGFA. In fact, bevacizumab can normalize blood vessels restoring DCs maturation and reducing Treg recruitment in CRC cells and MDSCs in renal cell carcinoma [38,39,40,41] (Figure 1).

## 3. Current Landscape of Anti-Angiogenetic Treatments in Metastatic CRC

### 3.1. Anti-Angiogenetic in First-Line Therapy

Anti-angiogenetic agents have an established role in the treatment of mCRC. In 2004, the phase 3 AVF2107g study proved that the addition of bevacizumab to backbone chemotherapy led to an improvement in both overall survival (OS) (20.3 vs. 15.6 months, HR 0.66; *p* < 0.001) and progression-free survival (PFS) (10.6 vs. 6.2 months, HR 0.66; *p* < 0.001) [4]; thus, bevacizumab was approved as the first targeted therapy for patients with mCRC. A subsequent systematic review and pooled analysis of 29 clinical trials investigating the efficacy of the combination FOLFIRI-bevacizumab as first-line treatment yielded similar results, with a RR of 51.4% (22 publications), a median PFS (25 publications) of 10.8 months (95% C.I. 8.9–12.8), and a median OS (20 publications) of 23.7 months (95% C.I., 18.1–31.6) [42].

Adding bevacizumab to fluorouracil/leucovorin, Folfox, and Xelox as first-line therapy proved similarly effective [43,44,45,46]. Kabbinavar et al. randomly assigned 104 previously untreated mCRC patients to fluorouracil/leucovorin alone or associated with bevacizumab at 5 mg/kg or 10 mg/kg [44]. The combination treatment with bevacizumab resulted in better response rates (RR) (17% in the control arm vs. 40% and 8% in the two bevacizumab arms, low and high doses), with longer mPFS (5.2 months vs. 9.0 and 7.2 months) and mOS (13.8 vs. 21.5 and 16.1 months, respectively) [44]. A subsequent trial comparing FU/LV plus bevacizumab/placebo and a combined analysis of three clinical studies [4,5] validated these data. The BECOME study assessed the effects of the addition of bevacizumab to mFOLFOX6 as a first-line treatment of the RAS mutant mCRC with unresectable liver metastases; a significant benefit was demonstrated for mOS (25.7 vs. 20.5 months; *p* = 0.03), mPFS (9.5 vs. 5.6 months; *p* = 0.01), ORR (54.5% vs. 36.7%; *p* < 0.01), and R0 resection rates for liver metastases (22.3% vs. 5.8%, *p* < 0.01) [45]. In a different randomized phase III trial, bevacizumab/placebo was evaluated associated with either FOLFOX4 or XELOX; mPFS was 9.4 months in the bevacizumab group and 8.0 months in the placebo group (HR 0.83; 97.5% C.I. 0.72–0.95; *p* = 0.0023), and median OS was 21.3 vs. 19.9 months (HR 0.89; 97.5% C.I., 0.76 to 1.03; *p* = 0.077); RR was similar across groups of treatment [46]. Different meta-analyses have, since then, confirmed a benefit in RR, PFS, and OS by adding bevacizumab to a combination chemotherapy [47,48,49,50,51,52,53].

With the approval, over the years, of different targeted therapies (most commonly anti-EGFR antibodies for RAS wild-type cancers), the need to define the optimal strategy led to different trials; the FIRE-3 trial is well known, which compared FOLFIRI-bevacizumab with FOLFIRI-cetuximab in the first-line setting for RAS wild-type patients, demonstrating a similar mPFS (10.0 vs. 10.3 months in the cetuximab and in the bevacizumab group, respectively; HR 1.06, 95% C.I. 0.88–1.26; *p* = 0.55) but a mOS in favor of the cetuximab group (28.7 vs. 25.0 months, HR 0.77, 95% C.I. 0.62–0.96; *p* = 0.017) [54]. The Calgb/Swog 80405 trial compared either FOLFOX or FOLFIRI plus bevacizumab or cetuximab, with a subsequent amendment to include only RAS wild-type patients; it showed an equivalence between regimens in terms of OS (29.0 vs. 30.0 months for the chemotherapy/bevacizumab and chemotherapy/cetuximab combinations, respectively; HR 0.88; 95% C.I., 0.77–1.01; *p* = 0.08) and PFS (10.6 vs. 10.5 months, respectively) [55]. The PEAK trial compared the addition of either panitumumab or bevacizumab in a population of patients with KRAS exon 2 wild-type cancers; PFS was similar, and OS was improved (41.3 vs. 28.9 months, HR, 0.63; 95% C.I., 0.39 to 1.02; *p* = 0.058) in the panitumumab compared to the bevacizumab arm [56]. The PARADIGM trial compared mFOLFOX6 with either panitumumab to bevacizumab in a KRAS/NRAS wild-type population; the trial is currently completed and pending results [57].

Moreover, bevacizumab has shown efficacy in association with a non-fluoropyrimidine-based chemotherapy regimen, such as in the phase III trial TRICOLORE, which compared an association of S-1 and irinotecan plus bevacizumab to a standard oxaliplatin-based chemotherapy regimen (mFOLFOX6 or CAPOX) associated with bevacizumab; results of the trial showed the non-inferiority of the trial regimen in regard to PFS (10.8 months in the control group and 14.0 months in the experimental group; HR 0.84, 95% C.I. 0.70–1.02; *p* < 0.0001 for non-inferiority, and *p* = 0.0815 for superiority) [58].

Bevacizumab has also been proven effective in association with the chemotherapy triplet FOLFOXIRI, in particular for the BRAF-mutant population [59,60,61]. In the phase II OLIVIA study, the triplet chemotherapy associated with bevacizumab was found to induce better response rates (81% vs. 62%) and improved mPFS (18.6 vs. 11.5 months); the TRIBE study confirmed these results [59].

In the elderly population, the AVEX study showed that bevacizumab in association with capecitabine induces a significantly longer PFS than capecitabine alone (9.1 vs. 5.1 months; HR 0.53 [0.41–0.69]; *p* < 0.0001) [62].

Finally, Holch et al. demonstrated the relevance of primary tumor location; in right-sided mCRC, bevacizumab appears to be the targeting agent of choice [63]. Even in left-sided RAS wild-type cancers, a combination of a triplet with bevacizumab appears at least non-inferior to FOLFOX-panitumumab [64].

Other antiangiogenetic agents, such as aflibercept, have been investigated in the first-line setting; in the phase III trial AFFIRM, patients with mCRC were randomized to receive first-line therapy with mFOLFOX6 plus aflibercept (4 mg/kg) or mFOLFOX6 alone; however, the results showed no difference in PFS between treatment arms [65] (Table 1).

### 3.2. Maintenance

The role of maintenance therapy in CRC has been recognized, although, especially in RAS wild-type patients, there is still a lack of consensus regarding the optimal choice of targeted agent. The SAKK 41/06 trial explored the non-inferiority of continuation of single-agent bevacizumab versus no treatment, after a first-line chemotherapy combined with bevacizumab. The trial failed to demonstrate non-inferiority and showed increased treatment costs with no clinically meaningful benefit for the bevacizumab arm [66]. The PRODIGE-9 trial, similarly, explored bevacizumab maintenance versus no treatment in chemotherapy-free intervals after first-line induction treatment with FOLFIRI-bevacizumab; bevacizumab maintenance did not improve chemotherapy-free intervals, PFS, or OS [67].

More promising results were obtained with a strategy of de-escalation of treatment, rather than chemotherapy-free intervals; maintenance treatments with a combination of fluoropyrimidine and bevacizumab yielded more favorable results. The CAIRO-3 trial explored the combination of capecitabine and bevacizumab as maintenance after six cycles of treatment with a combination of capecitabine, oxaliplatin, and bevacizumab [68]. Patients in the treatment arm had a significant improvement of time to second progression, which was the primary endpoint of the study (11.4 months in the observation group vs. 13.9 months in the maintenance group; HR 0.63, 95% C.I. 0.52–0.76, *p* < 0.0001). The non-inferiority phase 3 trial AIO 0207 compared no treatment and treatment with bevacizumab alone to a maintenance with capecitabine and bevacizumab. Regarding the primary endpoint, which was time to failure of strategy, bevacizumab alone was non-inferior to standard fluoropyrimidine plus bevacizumab (HR 1.08, 95% C.I. 0.85–1.37; *p* = 0.53; upper limit of the one-sided 99.8% CI 1.42), whereas no treatment was not (HR 1.26, 95% C.I. 0.99–1.60, *p* = 0.056; upper limit of the one-sided 99.8% C.I. 1.65); to note, only 36% of patients received a re-induction as per protocol, thus limiting the clinical value of the chosen primary endpoint. However, in terms of PFS, pairwise comparison between treatment groups showed a significant improvement for the more active treatment group for each comparison, thus favoring the combination of capecitabine/bevacizumab [69] (Table 2).

### 3.3. Second Line

The E3200 trial is a phase III trial that explored the efficacy of bevacizumab as a second-line treatment in patients with previously treated mCRC; in the trial, patients who received a first-line treatment with fluoropyrimidines and irinotecan were randomized to treatment with either FOLFOX4 alone, FOLFOX4 in combination with bevacizumab, or bevacizumab alone [70]. The median OS was 12.9 months for the combination arm, compared with 10.8 months for the FOLFOX4 group and 10.2 months for those treated with bevacizumab alone. The median PFS was 7.3 months for the combination group, compared with 4.7 months for the FOLFOX4 group and 2.7 months for the bevacizumab group. Overall RR were, respectively, 22.7%, 8.6%, and 3.3% (*p* < 0.0001 for FOLFOX4/bevacizumab vs. FOLFOX4) [70].

Continuation of bevacizumab beyond progression has been investigated by different trials. The ML18147 trial assessed the efficacy of continuation of bevacizumab associated with a second-line chemotherapy after progression on a standard first-line of chemotherapy associated with bevacizumab [71]. mOS was 11.2 months for the bevacizumab arm, compared to 9.8 months for chemotherapy alone (HR 0.81, 95% C.I. 0.69–0.94; *p* = 0.0062) [71]. The BEBYP trial, which investigated the same therapeutic strategy [34], confirmed these findings, showing a significant improvement in PFS for the bevacizumab group (5.0 vs. 6.8 months, adjusted HR 0.70; 95% C.I. 0.52–0.95; stratified log-rank *p* = 0.010) and a trend toward an improved OS (adjusted HR 0.77; 95% C.I. 0.56–1.06; stratified log-rank *p* = 0.043); the study was prematurely stopped in consideration of the results of the ML18147 trial [72]. The EAGLE trial explored two different doses of bevacizumab (5 or 10 mg/kg) associated with FOLFIRI as second-line therapy after progression on a oxaliplatin/bevacizumab-based first line, finding no benefits associated with the use of a higher dose [73]. Analysis from the ARIES observational cohort confirmed the observed improvement in PFS (14.4 months vs. 10.6 months) [74].

The use of different antiangiogenetic agents has been explored by the VELOUR and the RAISE trials. The VELOUR phase III study examined the addition of aflibercept to a FOLFIRI regimen after progression in an oxaliplatin-based first-line treatment; the study allowed for bevacizumab as part of the first-line regimen [5]. The experimental arm demonstrated an improvement of mOS (13.5 vs. 12.06 months, HR 0.8; 95.34% C.I., 0.71–0.937; *p* = 0.0032), mPFS (6.9 vs. 4.67 months; HR, 0.758; 95% C.I. 0.66–0.86; *p* < 0.0001), and RR (19.8% vs. 11.1%, *p* = 0.0001); this benefit was consistent across subgroups, including the patients who received bevacizumab as part of their first-line therapy [5]. The RAISE trial explored the combination of FOLFIRI with either ramucirumab of placebo after a first-line treatment with bevacizumab, oxaliplatin, and a fluoropyrimidine; results demonstrated an improvement in mOS for the ramucirumab group over the placebo group (13.3 months vs. 11.7 months, HR 0.84, 95% C.I. 0.73–0.97; log-rank *p* = 0.0219) [7] (Table 3).

### 3.4. Beyond Second-Line Treatment

Use of antiangiogenetics beyond second-line treatment has been proven effective by several clinical trials. In a phase II study, the addition of bevacizumab to a standard treatment with TAS-102 in chemo-refractory patients induced an improvement in PFS (4.6 vs. 2.6 months; HR 0.45; *p* = 0.0015); these results were recently confirmed by the phase III SUNLIGHT trial, where the combination treatment showed an improvement of both mOS (10.8 vs. 7.5 months; HR, 0.61; 95% C.I., 0.49–0.77; *p* < 0.001) and mPFS (5.6 vs. 2.4 months; HR 0.44; 95% C.I., 0.36–0.54; *p* < 0.001) [75,76].

Regorafenib, a multi-kinase inhibitor, targets angiogenic (VEGFR-1-3, TIE2), stromal (PDGFR-β, FGFR), and oncogenic receptor tyrosine kinases (KIT, RET, and RAF). Following the results of the CORRECT trial, which showed an improvement in OS (6.4 vs. 5.0 months, HR 0.77; 95% C.I. 0.64–0.94; *p* = 0.0052) for treatment with regorafenib in chemo-resistant patients when compared to placebo, this drug was approved as a monotherapy for mCRC patients who progressed on previous lines of treatment [6]. Similar results were shown in the Asian population in the CONCUR trial (mOS 8.8 vs. 6.3 months; HR 0.55, 95% C.I. 0.40–0.77, *p* = 0.00016) [77] (Table 4).

## 4. Looking to the Future

### 4.1. New Treatment Goals in CRC

Mounting evidence in tumor biology have shown that understanding the various mechanisms behind tumor growth and progression requires a comprehensive investigation of the complex interactions between tumor cells and their TME in order to also use them as targets for active and effective therapies to be used in clinical practice.

In addition, TME harbors multiple actors that practically play a significant role in tumor initiation, progression, and metastatization as the genetic and epigenetic changes in cancer cells. As already written, there is an important connection between angiogenesis, hypoxia, and immune response. These premises have paved the way for testing new combinations and new molecules in order to control cancer progression.

### 4.2. Immunotherapy and Anti-Angiogenics

Over the last years, immunotherapy has reshaped the landscape in several tumors, both as a single agent and combined with chemotherapy, like melanoma, lung, and urothelial cancer, improving survival outcomes.

Based on these premises, many clinical trials have been designed to revert to the immune-suppressive microenvironment driven by vasculopathy, exploiting the interplay between angiogenesis and the immune system. In fact, the restoration of immune responsiveness induced by combining anti-angiogenic therapy with immunotherapy could reverse the TME balance towards an immune-activated system (Figure 1).

Current clinical practice mainly includes three types of immune checkpoint inhibitors (ICIs) targeting CTLA4, PD-1, or PD-L1 [78]. Unfortunately, these treatments as single agents did not achieve sufficient data in CRC, except for microsatellite instability (MSI) tumors [79].

Moreover, in more recent years, as the immune-suppressive TME is induced in part by the abnormal vasculature, the combination with antiangiogenetics has been tested in several tumors like NSCLC (atezolizumab plus bevacizumab), renal cell carcinoma (cabozantinib plus nivolumab or axitinib plus pembrolizumab), HCC (atezolizumab plus bevacizumab), and endometrial cancer (lenvatinib plus pembrolizumab), improving survival outcomes [80,81,82,83].

This broad efficacy has been the key premise for transferring this combination treatment also in CRC. Firstly, Bendel et al. in 2015 presented an improved objective tumor response among patients receiving bevacizumab with atezolizumab at the ASCO-GI conference [84]. Likewise, bevacizumab plus atezolizumab was added to chemotherapy as a first-line treatment administered to 23 CRC patients and showed a median PFS of 14.1 months [85].

The first large trials were presented by Grothey et al. Overall, 445 BRAF wild-type CRC patients included in the MODUL trial were randomly assigned to receive maintenance therapy with fluoropyrimidine and bevacizumab with or without atezolizumab after induction first-line treatment with FOLFOX plus bevacizumab. However, no differences between the two arms were reported in terms of PFS and OS [86,87]. Biomarker analyses are still ongoing.

Mettu et al. presented at ESMO 2019 results derived from the BACCI phase II trial conducted on 133 CRC patients treated at last-line with capecitabine plus bevacizumab and atezolizumab or placebo, meeting the primary endpoint; however, the benefit was only 1 month [88]. In the atezolizumab arm, mPFS was 4.4 months vs. 3.6 months in the control arm. An exploratory analysis observed a higher ORR in patients without liver disease (23.1% vs. 5.8%), accompanied by a better PFS and OS [88].

The updated results from the phase Ib trial REGONIVO provide an encouraging antitumor activity in the enrolled CRC patients receiving nivolumab 3 mg/kg every two weeks combined with regorafenib. Median PFS was 7.8 months, 1y-PFS was 41.7%, and 1y-OS was 68% [89]. These findings paved the way for other clinical trials investigating the combination of anti-VEGF with immune checkpoint blockade.

From 2018 to 2020, 218 CRC patients were randomized 1:2, in the phase II ATEZOTRIBE trial, to receive first-line FOLFOXIRI plus bevacizumab with or without atezolizumab, followed by maintenance treatment (fluorouracil and leucovorin plus bevacizumab with or without atezolizumab), irrespective of RAS and BRAF mutational status [90]. Median PFS was 13.1 months in the experimental arm compared to 11.5 months in the control one (HR 0.69, 80% C.I. 0.56–0.85, *p* = 0.012). OS data are still immature. The most, 3–4, adverse events were neutropenia (42% vs. 36% in the experimental and control group, respectively), diarrhea (15% vs. 13%), and febrile neutropenia (10% vs. 10%) [90]. This trial suggested that a synergic role is played by combining atezolizumab with first-line chemotherapy plus anti-VEGF. Additionally, in the translation exploratory analysis, patients with high tumor mutational burden (TMB) and high immunoscore demonstrated a longer PFS [90].

The CheckMate 9X8 investigated the combined first-line FOLFOX plus bevacizumab, added or not to nivolumab, regardless of mutational status [91]. The primary endpoint was not achieved. Median PFS was 11.9 months in both groups. However, authors described a higher ORR (60% vs. 46%) and prolonged duration of response (DOR) in patients treated with nivolumab compared to standard treatment. Similarly, the NIVACOR phase II single-arm study tested first-line FOLFOXIRI plus bevacizumab and nivolumab, followed by a maintenance strategy in RAS or BRAF mutant CRC. ORR was 76.7% in the whole population and 78.9% in the MSS mCRC patients, while median PFS was 9.8 months [92].

In the NCT03050814 phase II trial, patients with MSS mCRC were randomized to FOLFOX plus bevacizumab ± avelumab with a CEA-targeted vaccine. No positive results have been obtained in terms of PFS [93]. Furthermore, toripalimab (a PD-1 antibody) added to regorafenib showed a good response and OS in a phase 2 study [94].

Main clinical trials evaluating anti-angiogenic drugs associated with ICIs are reported in Table 5.

### 4.3. Targeting Angiogenesis Through a New Generation Molecule

In recent years, a new generation molecule, fruquintinib, highly selective for VEGFR-1, 2, and 3, has been developed based on the great antitumor activity demonstrated both in in vitro and in vivo models [95]. It exerts its properties by suppressing endothelial cell proliferation and tubule development in a dose-dependent manner. The phase III FRESCO trial randomly enrolled Chinese patients after two lines of therapy to receive fruquintinib at 5 mg administered on days 1–21 every 4 weeks plus BSC, or placebo plus BSC [96]. The experimental arm significantly improved the survival outcome, gaining a longer OS (9.3 vs. 6.6 months; HR 0.65, 95% C.I. 0.51–0.83, *p* < 0.001) and PFS (3.71 vs. 1.84 months; HR 0.26, 95% C.I. 0.21–0.34, *p* < 0.001) with an acceptable safety profile [96]. Based on these data, fruquintinb received its approval in China on 4 September 2018. However, given geographic differences between China and the Western population, a global phase III study has been developed: the FRESCO-2 trial [97].

Heavily pre-treated patients (at least two lines) were randomized 2:1 to receive fruquintinib plus BSC or placebo plus BSC. Consistently, the FRESCO-2 trial reached its primary and secondary endpoints, confirming the OS (7.4 vs. 4.8 months; HR 0.66, 95% C.I. 0.55–0.80, *p* < 0.001) and PFS (3.7 vs. 1.8 months; HR 0.32, 95% C.I. 0.27–0.39, *p* < 0.001) improvement.

The OS benefit has been demonstrated across all subgroups, including patients treated with TAS-102, regorafenib, or both (HR 0.60) [97].

Ongoing clinical trials are exploring the optimal strategy to use fruquintinib in mCRC patients, in first-line (NCT01975077), second-line (NCT05634590, NCT05555901, NCT05522738, and NCT05447715), or third-line (NCT05447715) combined with chemotherapy or with TAS-102 (NCT05004831). Further, in chemo-refractory patients, fruquintinb added to immunotherapy or raltitrexed is being tested (NCT04695470, NCT04582981, and NCT04866862) (Table 6).

## 5. Identification of Prognostic and Predictive Biomarkers During Anti-Angiogenic Treatment

The key role played by angiogenesis in tumor growth and spreading is well established. However, not all patients seem to benefit from anti-angiogenic treatment. Henceforth, the identification of prognostic and predictive biomarkers is necessary to better refine mCRC patients who will be more likely to benefit from these therapies.

Several potential biomarkers have been investigated as implicated in angiogenesis. First of all, circulating VEGFR analysis has been conducted in many trials with contrasting results. A subgroup of 59 patients enrolled in the BEBYP trial has been tested for VEGFR-2 levels, showing that in the group with high VEGFR-2 (>median value, 6.3 ng/mL), the prosecution of bevacizumab was associated with a better PFS (median PFS: 10.4 vs. 3.4 months; HR 0.24, 95% C.I. 0.10–0.58, *p* = 0.002). However, the benefit from the prosecution of bevacizumab was not reported in patients with low VEGFR-2 levels (mPFS 5.4 vs. 5.0; HR 0.98, 95% C.I. 0.45–2.11, *p* = 0.956) [98].

VEGF-D has been dosed in the MAX, CAIRO-2, and RAISE studies. In the first one, low expression of VEGF-D was associated with efficacy of bevacizumab-based therapy regarding PFS (HR 0.21, 95% C.I. 0.08–0.55) and OS (HR 0.35; 95% C.I. 0.13–0.90). Less efficacy was observed in the population with low VEGF-D levels. In CAIRO-2, no differences have been reported according to VEGF-D levels [99]. In the RAISE trial, ramucirumab added to chemotherapy resulted in more effective outcomes in the group with high VEGF-D expression, both for PFS and OS [100]. VEGF-A showed no predictive role in different retrospective and prospective studies, while VEGF-A splice isoform 165b and 121 seem to be a potential predictive biomarker of response to bevacizumab [101,102,103]. Preclinical studies observed an important role played by FGF-2 in restoring sensitivity during bevacizumab treatment. Giampieri et al. observed a significant association of high FGF2 expression with a longer PFS and OS, despite high FGF-2 levels at baseline representing a poor prognostic factor [104].

Further, preclinical studies observed a correlation between serum LDH levels with VEGF-A and VEGFR-1 expression [105]. High serum LDH levels seem to be associated with benefit from VEGF-A inhibition. However, the analysis conducted on the BEBYP trial revealed contrasting results; bevacizumab beyond progression was effective in the group with low LDH levels (HR: 0.39, 95% C.I.: 0.23–0.65), while no difference was reported in the group with high LDH levels HR: 1.10, (95% C.I.: 0.74–1.64) [106,107,108].

Many biomarkers have been identified as predictive factors during anti-angiogenic treatment, such as the transcription factor homeobox 9, hepatocyte growth factor, and markers of vascular immaturity [100,103,109,110,111]. The loss of chromosome 18q11.2–q12 [112] has been studied in a non-randomized trial and, then, in a post hoc analysis of the 256 AGIT-MAX trial. This loss was detected in 71% of mCRC patients and was associated with a bevacizumab benefit in terms of PFS (*p* = 0.009) and not in patients without (*p* = 0.67). However, a statistical significance for marker–treatment interaction was not yielded (*p* = 0.28) [113]. Moreover, several miRNAs have been tested to demonstrate their association with outcomes during anti-angiogenic treatment. High miR-664-3p levels were significantly associated with better survival in mCRC patients treated with bevacizumab compared to the placebo group [114]. Hamaguchi et al. conducted an exploratory analysis evaluating 78 potential prognostic and predictive biomarkers in 62 Japanese patients receiving aflibercept plus chemotherapy. Among all biomarkers, low levels of an extracellular newly identified receptor for advanced glycation end-products binding protein (EN-RAGE), tissue inhibitor of metalloproteinases 1 (TIMP-1), and interleukin-8 (IL-8) allowed for a beneficial gain in terms of OS [115,116]. Nonetheless, these biomarkers have not demonstrated sufficient prognostic and predictive data to warrant their translation into current clinical practice, even if promising. Hence, further prospective analyses should be conducted to better identify a class of patients that could benefit from any anti-angiogenic treatment.

## 6. Cost-Effectiveness of Anti-Angiogenic Treatment

Due to the lack of predictive biomarkers, the antiangiogenic-based treatments are broadly used, at least once, during the treatment history of mCRC patients. Hence, since it is administered in an unselected population and only some fraction of patients will really benefit from it with a considerable economic burden, a cost-effectiveness balance is mandatory.

The high variability of economic policies and pricing in individual countries is relevant for the cost-effectiveness analysis. However, the addition of anti-angiogenic therapy in mCRC is not cost-effective in most countries. In the analysis conducted by Goldstein et al. in 2017, the addition of bevacizumab to first-line chemotherapy in mCRC consistently failed to be cost-effective in all five countries (the U.S., the U.K., Australia, Canada, and Israel) evaluated, with the highest incremental cost-effectiveness ratio for the U.S. (USD 571,000 per quality-adjusted life years) [117]. Similar results are reported for bevacizumab in the maintenance strategy. Moreover, the introduction of anti-angiogenic drugs in second-line treatment was linked with a significant increase in costs. An analysis conducted on four randomized clinical trials, including 3938 mCRC patients treated with anti-angiogenic second-line therapy, combined the pharmacological costs of the drug with the benefit measured according to PFS. FOLFIRI plus aflibercept was the most cost-effective treatment with the lowest cost per month of PFS (EUR 4581) [118]. Further analyses will need to be conducted to establish the full balance between costs and benefits deriving from the newly developed molecule and, particularly, from the combination of anti-angiogenic therapies with immunotherapy.

## 7. Expert Opinion and Future Directions

Angiogenesis has long been recognized as a fundamental hallmark of cancer progression, especially in mCRC. Since the first approval in 2004 of bevacizumab, with encouraging results, several anti-angiogenic agents have been developed for the treatment of mCRC in the last few decades: ziv-aflibercept and regorafenib in 2012, and ramucirumab in 2014. Although most pivotal trials on antiangiogenic therapy in mCRC were conducted before 2018, they remain the key evidence supporting current treatment strategies. More recent studies have focused on biomarker identification, treatment sequencing, and combinations with immunotherapy, rather than introducing new antiangiogenic agents. More recently, a new molecule that is highly selective for VEGFR-1, 2, and 3, fruquintinb, showed its efficacy in improving mCRC prognosis. It exerts its properties by reducing endothelial cell proliferation and tubule growth. The FRESCO-2 trial showed the PFS and OS benefit in heavily pre-treated CRC patients.

Interestingly, the abnormal architecture of tumor blood vessels enhances the intra-tumoral pressure and decreases the endothelial intracellular adhesion molecules. Likewise, hypoxia and acidosis induce the up-regulation of immune-suppressive chemokines that recruit several immune-suppressive cytokines, molecules, and cells, such as Treg and MDSCs, into the tumor bed, polarize macrophages towards the M2-like phenotype, and reduce the extravasation of TILs. The use of anti-angiogenetic agents could reverse the immunosuppressive microenvironment. Thus, as the abnormal vessels can affect TME, combination treatments based on antiangiogenics and immune checkpoint inhibitors have also been tested in mCRC. In fact, the first clinical data with immunotherapy only became available in 2015, with initial limitation to MSI-high mCRC. Mounting data have recently demonstrated the synergic role played by the combination of immunotherapy with anti-VEGF.

According to the data discussed in the present narrative review, the therapeutic landscape of mCRC is likely to change in the near future.

However, it raises several challenges and some open questions.

The first is “positioning”: What will be the optimal strategy in the treatment of mCRC? Many molecules and combination treatments, in addition to anti-angiogenic drugs, are being tested in the mCRC treatment. Furthermore, ongoing clinical studies are evaluating the best strategy for the use of fruquintinib in mCRC patients, in first-, second-, or third-line combined with chemotherapy or immunotherapy. Moreover, many clinical trials are evaluating the best treatment combinations between anti-angiogenic drugs and immune checkpoint inhibitors. So, how will these therapy options fit into future mCRC scenarios?

Secondly, how do we identify patients who can benefit from anti-angiogenic agents?

Many doubts persist regarding patient selection, optimal sequencing, and the integration of antiangiogenics within a modern treatment algorithm that increasingly emphasizes molecular stratification and immunologic modulation.

In fact, not all CRC patients benefit from treatment with angiogenic inhibitors alone or combined with immunotherapy. However, none of the biomarkers tested (VEGFR-2, VEGF-D, FGF-2, LDH, EN-RAGE, TIMP-1, IL-8, transcription factor homeobox 9, the loss of chromosome 18q11.2–q12, VEGFR-1, and VEGF-A) have demonstrated sufficient prognostic and predictive information to warrant their use in current clinical practice. Hence, further prospective analyses should be conducted to better identify how, when, and the class of mCRC patients that could more likely benefit from these therapies.

Thirdly, what is the balance between cost and efficacy of the new proposed strategy? Different cost-effective analyses have been conducted regarding the use of bevacizumab and aflibercept. However, further analyses will need to be conducted to establish the full balance between costs and benefits deriving from this newly developed molecule and, particularly, from the combination of anti-angiogenic therapies with immunotherapy.

Antiangiogenic therapy remains an important player in mCRC treatment. However, to preserve its role in the evolving treatment landscape, a paradigm shift is needed. Combinations with immunotherapy seem to be a great opportunity for mCRC, but understanding the interplay of the tumor immune microenvironment and angiogenic dynamics is necessary to better define responsive patients. Despite extensive available data, no predictive biomarkers have yet demonstrated sufficient reliability or validation for routine clinical application in mCRC patients treated with antiangiogenic agents. Hence, ongoing research should prioritize biomarker discovery, rational combinations, and patient stratification to optimize the clinical impact and economic viability of antiangiogenic agents in mCRC.

## Figures and Tables

**Table 1:** First-line trials.

Citation	Trial	N° pt	Treatment	Type of Trial	ORR	PFS	OS
Hurwitz et al., NEJM 2004 [4]	AVF2107g	813	Irinotecan/leucovorin/fluorouracil + Bevacizumab/placebo	Phase III, blinded, randomized	44.8% vs. 34.8% (*p* = 0.004)	10.6 vs. 6.2 m (*p* < 0.001)	20.3 vs. 15.6 m (*p* < 0.001)
Kabbinavar et al., JCO 2003 [43]	NA	104	Leucovorin/fluorouracil + placebo/Bevacizumab low dose/Bevacizumab high dose	Phase II, open-label, randomized	17% vs. 40% vs. 24%	5.2 vs. 9.0 vs. 7.2 m	13.8 vs. 21.5 vs. 16.1 m
Kabbinavar et al., JCO 2005 [44]	NA	209	Leucovorin/fluorouracil + bevacizumab/placebo	Phase II, blinded, randomized	26% vs. 15.2% (*p* = 0.055)	9.2 vs. 5.5 m (*p* = 0.0002)	16.6 vs. 12.9 m (*p* = 0.16)
Tang W et al., JCO 2020 [45]	BECOME	241	mFOLFOX6 +/− Bevacizumab	Phase IV, open-label, randomized	54.5% vs. 36.7% (*p* < 0.01)	9.5 vs. 5.6 m (*p* < 0.01)	25.7 vs. 20.5 m (*p* = 0.03)
Saltz et al., JCO 2008 [46]	NO16966	1401	XELOX/FOLFOX4 + bevacizumab/placebo	Phase III, blinded, randomized	47% vs. 49% (*p* = 0.31)	9.4 vs. 8 m (*p* = 0.0023)	21.0 vs. 19.9 m (*p* = 0.077)
Schmiegel et al., Ann Oncol 2013 [8]	NA	255	Bevacizumab + Capox/mCapIri	Phase II, randomized, open-label	53% vs. 56%	10.4 vs. 12.1 m	24.4 vs. 25.5 m
Heinemann V et al., Lancet Onc 2014 [54]	FIRE 3	592	FOLFIRI + Cetuximab/Bevacizumab	Phase III, randomized, open-label	62% vs. 58% (*p* = 0.18)	10.0 vs. 10.3 m (*p* = 0.55)	28.7 vs. 25.0 m (*p* = 0.017)
Venook et al., JAMA 2017 [55]	Calgb/Swog 80405	3058 (2334 KRAS WT)	FOLFIRI/mFOLFOX6 + cetuximab/bevacizumab	Phase III, randomized, open-label	59.6% vs. 55.2% (*p* = 0.13)	10.5 vs. 10.6 m (*p* = 0.45)	30.0 vs. 29.0 m (*p* = 0.08)
Schwartzberg et al., JCO 2014 [56]	PEAK	285	mFOLFOX6 + panitumumab/bevacizumab	Phase II, randomized, open-label	57.8% vs. 53.5%	10.9 vs. 10.1 m (*p* = 0.353)	34.2 vs. 24.3 m (*p* = 0.009)
Yamada et al., Ann Oncol 2018 [58]	TRICOLORE	487	mFOLFOX6/Capox + bevacizumab vs. S1 + Irinotecan + Bevacizumab	Phase III, randomized, open-label, non-inferiority	70.6% vs. 66.4% (*p* = 0.34)	10.8 vs. 14.0 m (*p* < 0.0001)	33.6 vs. 34.9 m (*p* = 0.2841)
Gruenberger et al., Ann Oncol 2015 [59]	OLIVIA	80	Bevacizumab + mFOLFOX6/FOLFOXIRI	Phase II, randomized, open-label	62% vs. 81%	11.5 vs. 18.0 m (HR 0.43)	32.2 vs. NR (HR 0.35)
Cremolini et al., Lancet Oncol 2015 [60]	TRIBE	508	Bevacizumab + FOLFIRI/FOLFOXIRI	Phase III, randomized, open-label	54% vs. 65% (*p* = 0.013)	9.7 vs. 12.3 m (*p* = 0.01)	25.8 vs. 29.8 m (*p* = 0.03)
Cunningham et al., Lancet Oncol 2013 [62]	AVEX	280	Capecitabine +/− Bevacizumab	Phase III, randomized, open-label		9.1 vs. 5.1 m (*p* < 0.0001)	20.7 vs. 17.0 m (*p* = 0.13)

**Table 2:** Maintenance trials.

Citation	Trial	N° pt	Treatment	Type of Trial	PFS	OS
Koeberle et al., Ann Oncol 2015 [66]	SAKK 41/06	262	Bevacizumab vs. no treatment	Phase III, randomized, open-label	4.1 vs. 2.9 m (TTP)	25.4 vs. 23.8 m (*p* = 0.2)
Aparicio et al., JCO 2018 [67]	PRODIGE 9	491	Bevacizumab vs. no treatment	Phase III, randomized, open-label	9.2 vs. 8.9 m (*p* = 0.316)	21.7 vs. 22.0 m (*p* = 0.5)
Goey et al., Ann Oncol 2017 [68]	CAIRO 3	558	Capecitabine/Bevacizumab vs. no treatment	Phase III, randomized, open-label	8.5 vs. 4.1 m (*p* < 0.0001)	21.6 vs. 18.2 (*p* = 0.1)
Hegewisch-Becker et al., Lancet Oncol 2015 [69]	AIO 0207	472	5FU/Bevacizumab vs. Bevacizumab vs. no treatment	Phase III, randomized, open-label, non-inferiority	6.3 vs. 4.6 vs. 3.5 m (*p* < 0.0001)	20.2 vs. 21.9 vs. 23.1 (*p* = 0.77)

**Table 3:** Second-line trials.

Citation	Trial	N° pt	Treatment	Type of Trial	PFS	OS
Giantonio et al., JCO 2007 [70]	E3200	829	FOLFOX4/Bevacizumab vs. FOLFOX4 vs. Bevacizumab	Phase III, open-label, randomized	7.3 vs. 4.7 vs. 2.7 m (*p* < 0.0001)	12.9 vs. 10.8 vs. 10.2 (*p* = 0.0011)
Bennouna et al., Lancet 2013 [71]	ML18147	409	Chemotherapy +/− Bevacizumab	Phase III, open-label, randomized	5.7 vs. 4.1 m (*p* < 0.0001)	11.2 vs. 9.8 m (*p* = 0.0062)
Masi et al., Ann Oncol 2015 [72]	BEBYP	185	Chemotherapy +/− Bevacizumab	Phase III, open-label, randomized	6.8 vs. 5.0 m (*p* = 0.010)	15.5 vs. 14.1 m (*p* = 0.043)
Iwamoto et al., Ann Oncol 2015 [73]	EAGLE	387	FOLFIRI + Bevacizumab 5/10 mg/kg	Phase III, open-label, randomized	6.1 vs. 6.4 m (*p* = 0.676)	16.3 vs. 17.0 m (*p* = 0.667)
Van Cutsem et al., JCO 2012 [5]	VELOUR	1226	FOLFIRI + Aflibercept/placebo	Phase III, double-blind, randomized	6.9 vs. 4.67 m (*p* < 0.0001)	13.05 vs. 12.06 m (*p* = 0.0032)
Tabernero et al., Lancet Oncol 2015 [7]	RAISE	1072	FOLFIRI + Ramucirumab/placebo	Phase III, double-blind, randomized	5.7 vs. 4.5 m (*p* = 0.0005)	13.3 vs. 11.7 m (*p* = 0.0219)

**Table 4:** Beyond second-line trials.

Citation	Trial	N° pt	Treatment	Type of Trial	PFS	OS
Pfeiffer et al., Lancet Oncol 2020 [75]	NA	93	TAS-102 +/− Bevacizumab	Phase II, open-label, randomized	4.6 vs. 2.6 m (*p* = 0.0010)	9.4 vs. 6.7 m (*p* = 0.028)
Tabernero et al., JCO suppl 2023 [76]	SUNLIGHT	492	TAS-102 +/− Bevacizumab	Phase III, open-label, randomized	5.6 vs. 2.4 m (*p* < 0.001)	10.8 vs. 7.5 m (*p* < 0.001)
Grothey et al., Lancet 2013 [6]	CORRECT	760	Regorafenib/placebo	Phase III, quadruple masking, randomized	1.9 vs. 1.7 m (*p* < 0.0001)	6.4 vs. 5.0 m (*p* = 0.0052)
Li et al., Lancet Oncol 2015 [77]	CONCUR	204	BSC +/− Regorafenib	Phase III, double-blind, randomized	3.2 vs. 1.7 m (*p* < 0.0001)	8.8 vs. 6.3 m (*p* = 0·00016)

**Table 5:** Summary of main clinical trials evaluating anti-angiogenic drugs plus ICIs.

Trial	Phase	Years	N. of pts	Treatment	mPFS	mOS
NCT01633970	Ib	December 2014 through April 2017	Arm A (pre-treated): 13 Arm B (naïve): 26	Arm A: atezolizumab + bevacizumab Arm B: atezolizumab + FOLFOX + bevacizumab	NA	NA
MODUL (NCT02291289)	II	April 2015 through July 2019	445 (naïve, BRAF wt)	Maintenance bevacizumab +/− atezolizumab after FOLFOX + bevacizumab	Not met	NA
BACCI (NCT02873195)	II	September 2017 through June 2018	133 (pre-treated)	Capecitabine and bevacizumab + atezolizumab/placebo	4.4 vs. 3.6 mo	NA
NCT03239145	Ib		18 (MSS, pre-treated)	Pembrolizumab + trebananib	NA	9 mo
NCT03946917	Ib/II	March 2019 through January 2020	42 (MSS pre-treated)	Toripalimab + regorafenib	2.1 mo	15.5 mo
LEAP-005 (NCT03797326)	II	2019–2020	32 (MSS, pre-treated)	Pembrolizumab + lenvatinib	2.3 mo	7.5 mo
NCT04126733	II	October 2019 through January 2020	70 (MSS, pre-treated)	Nivolumab + regorafenib	15 weeks	52 weeks
REGOMUNE (NCT03475953)	II	November 2018 through October 2019	48 (MSS, pre-treated)	Avelumab + regorafenib	3.6 mo	10.8 mo
CheckMate 9X8 (NCT03414983)	II	February 2018 through April 2019	Experimental Arm: 127 Control Arm:68	Experimental Arm: FOLFOX + Bevacizumab + Nivolumab Control Arm: FOLFOX + Bevacizumab	11.9 mo in both arms	Immature data
Atezo TRIBE (NCT03721653)	2	November 2018 through February 2020	218 (naïve)	FOLFOXIRI + bevacizumab +/− atezolizumab	13.1 vs. 11.5 mo (*p* = 0.012)	NA
NIVACOR (NCT04072198)	2	October 2019 through March 2021	73 (naïve, RAS/BRAF mut)	FOLFOXIRI + bevacizumab + nivolumab	10.1 mo	NA
NCT03396926	2	April 2018 through October 2021	44 (MSS, pre-treated)	Pembrolizumab + capecitabine + bevacizumab	4.3 mo	9.6 mo
NCT03050814	2	April 2017 through October 2019	26 (MSS, naïve)	mFOLFOX6 + bevacizumab +/− avelumab + CEA-targeted vaccine	No diff.	NA
NCT03712943	1b	November 2018 through September 2020	51 (MSS, pre-treated)	Nivolumab + regorafenib	4.3 mo	11.1 mo
NCT03657641	1/2	July 2019 through July 2021	73 (MSS, pre-treated)	Pembrolizumab + regorafenib	2.8 mo	9.6 mo

Legend: pts = patients; mPFS = median progression-free survival; mOS = median overall survival; MSS = microsatellite stability; MSI = microsatellite instability; mo = months.

**Table 6:** Summary of ongoing clinical trials with fruquintinb.

Trial	Phase	Study Start	Setting	Treatment	Primary Endpoint
NCT05004441	II	2021	First-line	FOLFOX/FOLFIRI, fruquintinib	ORR
NCT04296019, NCT05016869, NCT05451719, NCT04733963, NCT05659290	II or I/II	2021–2023	Maintenance	Fruquintinib or fruquintinib plus capecitabine	PFS
NCT05634590	II	2022	Second-line	FOLFOX/FOLFIRI, fruquintinib	PFS
NCT05555901	II	2023	Second-line	FOLFIRI plus fruquintinib vs. FOLFIRI plus bevacizumab	PFS
NCT05522738	Ib/II	2022	Second-line	FOLFIRI, fruquintinib	ORR
NCT05447715	II	2022	Second-/Third-line	Fruquintinib sequential bevacizumab plus FOLFIRI vs. bevacizumab plus FOLFIRI sequential fruquintinib	PFS
NCT05004831	II	2022	Third-line	Fruquintinib, trifluridine/tipiracil	PFS
NCT04695470	II	2020	Pre-treated	Fruquintinib, sintilimab	PFS
NCT04582981	II	2020	Pre-treated	Fruquintinib plus raltitrexed vs. fruquintinib	PFS
NCT04866862	II	2021	Pre-treated	Fruquintinib, camrelizumab	ORR

Legend: PFS = Progression-free survival; ORR = Overall response rate.

## Data Availability

No new data were created or analyzed in this study. Data sharing is not applicable to this article.

## Abbreviations

BRAF	v-Raf murine sarcoma viral oncogene homolog B
BSC	Best supportive care
CEA	Carcinoembryonic antigen
CI	Confidence interval
CRC	Colorectal cancer
CTLA-4	Cytotoxic T-lymphocyte–associated protein 4
CXCL9/10	C-X-C motif chemokine ligand 9/10
DCs	Dendritic cells
DOR	Duration of response
ECM	Extracellular matrix
ECs	Endothelial cells
EGF	Epidermal growth factor
EGFR	Epidermal growth factor receptor
EMA	European Medicines Agency
EN-RAGE	Extracellular newly identified receptor for advanced glycation end-products binding protein
ESMO	European Society for Medical Oncology
FDA	U.S. Food and Drug Administration
FGF-2	Fibroblast growth factor-2
FGFR	Fibroblast growth factor receptor
FOLFIRI	5-Fluorouracil + leucovorin + irinotecan
FOLFOX	5-Fluorouracil + leucovorin + oxaliplatin
FOLFOXIRI	5-Fluorouracil + leucovorin + oxaliplatin + irinotecan
FU/LV	5-Fluorouracil/leucovorin
HCC	Hepatocellular carcinoma
HGF	Hepatocyte growth factor
HIF-1α	Hypoxia-inducible factor-1 alpha
HR	Hazard ratio
ICI(s)	Immune checkpoint inhibitor(s)
IL-8/IL-12/IL-18	Interleukin-8/-12/-18
INF-α/IFN-γ	Interferon-alpha/interferon-gamma
KD	(not used)
KIT	KIT proto-oncogene receptor tyrosine kinase
KRAS/NRAS	Kirsten rat sarcoma/neuroblastoma RAS viral oncogene homolog
LAG-3	Lymphocyte activation gene-3
LDH	Lactate Dehydrogenase
mCRC	Metastatic colorectal cancer
mFOLFOX6	Modified FOLFOX6 regimen
mOS	Median overall survival
mPFS	Median progression-free survival
MDSCs	Myeloid-derived suppressor cells
MVD	Microvascular density
MSS	Microsatellite atable
MSI/MSI-H	Microsatellite instability/microsatellite instability-high
mRNA	Messenger ribonucleic acid
NRP1/NRP2	Neuropilin-1/neuropilin-2
NSCLC	Non–small-cell lung cancer
ORR	Overall response rate
OS	Overall survival
PD-1	Programmed cell death-1
PD-L1	Programmed death-ligand 1
PDGF(s)	Platelet-derived growth factor(s)
PDGFR-β	Platelet-derived growth factor receptor-beta
PFS	Progression-free survival
PlGF	Placental growth factor
RAF	Rapidly accelerated fibrosarcoma kinase family
RAS	Rat sarcoma viral oncogene family (KRAS/NRAS)
R0	Microscopically margin-negative resection
RET	RET proto-oncogene
RR	Response rate
RTKs	Receptor tyrosine kinases
S-1	Tegafur/gimeracil/oteracil (oral fluoropyrimidine)
TAM(s)	Tumor-associated macrophage(s)
TAS-102	Trifluridine/Tipiracil
TCE	(not used)
TIE2	TEK tyrosine kinase endothelial receptor 2
TILs	Tumor-infiltrating lymphocytes
TIMP-1	Tissue inhibitor of metalloproteinases-1
TMB	Tumor mutational burden
TME	Tumor microenvironment
TNF-α	Tumor necrosis factor-alpha
Treg	Regulatory T cell
US	United States
VCAM-1	Vascular cell adhesion molecule-1
VEGF	Vascular endothelial growth factor
VEGF-A/-B/-C/-D	VEGF isoforms A/B/C/D
VEGFR-1/-2/-3	Vascular endothelial growth factor receptor-1/-2/-3
XELOX/CAPOX	Capecitabine + oxaliplatin
